# Paradoxical Para-Hisian Pacing Response

**DOI:** 10.19102/icrm.2023.14063

**Published:** 2023-06-15

**Authors:** Tolga Han Efe, Idriz Merovci, Muhammed Yunus Calapkulu, Ceren Ozdemir Al, Meryem Kara, Duygu Kocyigit Burunkaya, Elif Hande Ozcan Cetin, Ahmet Korkmaz, Ozcan Ozeke, Serkan Cay, Firat Ozcan, Dursun Aras, Serkan Topaloglu

**Affiliations:** ^1^Department of Cardiology, University of Health Sciences, Dışkapı Yıldırım Beyazıt Training and Research Hospital, Ankara, Turkey; ^2^Department of Cardiology, University Clinical Center of Kosovo, Prishtina, Kosovo; ^3^Department of Cardiology, University of Health Sciences, Ankara City Hospital, Ankara, Turkey; ^4^Department of Cardiology, İstanbul Medipol University, Istanbul, Turkey

**Keywords:** Atrioventricular re-entrant tachycardia, elecrophysiological maneuvers, para-Hisian pacing

## Abstract

Para-Hisian pacing (PHP) is among the most useful maneuvers in cardiac electrophysiology during sinus rhythm and identifies whether retrograde conduction is dependent on the atrioventricular (AV) node. In this maneuver, the retrograde activation time and pattern are compared during capture and loss of capture of the His bundle while pacing from a para-Hisian position. A common misconception about PHP is that it is useful only for septal accessory pathways (APs). However, even with left or right lateral pathways, as long as pacing from the para-Hisian region conducts to the atrium with the activation sequence being analyzed, it can be used to determine whether that activation is AV node–dependent or AP-dependent.

## Case presentation

**Figure 1:**
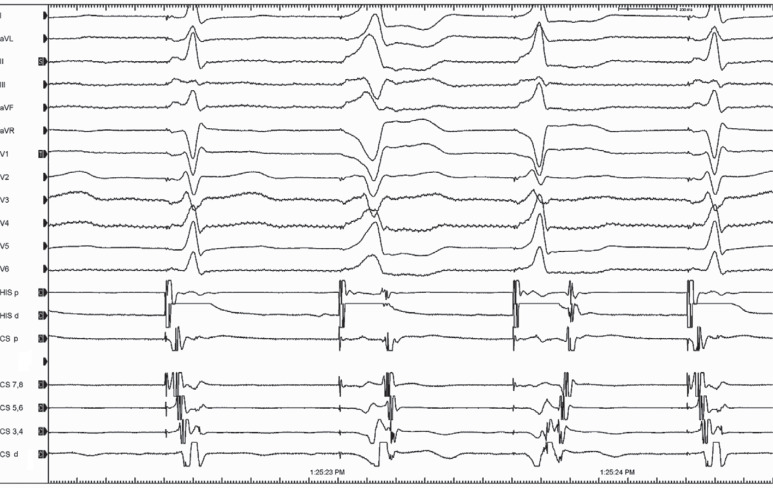
Three different QRS morphologies and ventriculoatrial intervals during para-Hisian pacing maneuver. *Abbreviations:* Cs d, coronary sinus distal; Cs p, coronary sinus proximal; His d, His distal; His p, His proximal.

A 29-year-old woman underwent an electrophysiology study (EPS) because of episodes of palpitations with documented narrow complex tachycardia (NCT). The electrocardiography during sinus rhythm (SR) did not exhibit any delta waves. During the EPS, the patient was in SR with normal atrio–His and His–ventricular intervals of 55 and 45 ms, respectively. Para-Hisian pacing (PHP) was performed to discriminate between a retrograde accessory pathway (AP) and atrioventricular (AV) nodal conduction **(Figure 1)**. Below, we consider what diagnostic information could be retrieved from this tracing.

## Discussion

PHP is one of the useful maneuvers in cardiac electrophysiology when performed and interpreted correctly.^1–8^ It is generally performed in the context of NCT with a central retrograde atrial activation (RAA) sequence where there is a question of AV node re-entry versus AV re-entry utilizing a septal AP. Therefore, it identifies essentially whether retrograde conduction is dependent on the AV node.^9,10^ It is an extremely useful maneuver, but careful attention must be paid to both the timing and activation sequence of the RAA. The RAA time and sequence are compared during capture and loss of capture of the His bundle while pacing from a para-Hisian position.^1–8^ When the stimulus–atrial (S–A) interval and RAA sequence are unchanged, this indicates retrograde conduction over an AP. When the S–A interval increases due to an identical increase of the stimulus–His interval with the same RAA sequence, this indicates retrograde conduction via an AV nodal pathway. In the current tracing, the S–A interval at the His catheter with wider QRS (second beat in **Figure 1**) was paradoxically shorter compared to that with narrow QRS (third beat in **Figure 1**).

It is important to understand certain limitations before concluding based on PHP.^5,8,11–24^ A common misconception about PHP is that it is useful only for septal APs.^25^ However, even with left or right lateral APs, as long as pacing from the para-Hisian region conducts to the atrium with the RAA sequence being analyzed, this maneuver can be used to determine whether that activation is AV node–dependent or AP-dependent. In the current tracing, the RAA sequence was converted from a concentric to an eccentric RAA sequence supporting the presence of the left lateral AP. How can the paradoxical situation be explained in this case? While the low-voltage pacing from the right ventricular para-Hisian myocardium was far away from the left lateral AP, the pacing from the same site with high voltage makes it closer to the left lateral AP. Therefore, at very high-output pacing, direct capture of the contralateral bundle (left bundle or left ventricular myocardium) can create earlier activation via a left-sided pathway, with high-output pacing giving a mistaken impression of retrograde AV node activation.^17,18,24^ After this extranodal response to the PHP maneuver, an orthodromic AV re-entrant tachycardia using the left posterolateral AP was diagnosed, and the posterolateral AP was ablated with satisfactory results.
